# Recombinant fowlpox virus vector-based vaccines: expression kinetics, dissemination and safety profile following intranasal delivery

**DOI:** 10.1099/jgv.0.000702

**Published:** 2017-04-01

**Authors:** David G Townsend, Shubhanshi Trivedi, Ronald J Jackson, Charani Ranasinghe

**Affiliations:** Molecular Mucosal Vaccine Immunology Group, Department of Immunology and Infectious Disease, The John Curtin School of Medical Research, The Australian National University, Canberra ACT 2601, Australia; ^‡^​ Present address: Division of Infectious Diseases, Department of Internal Medicine, The University of Utah, Salt Lake City, UT, USA.

**Keywords:** Fowlpox virus vectors, GFP, mCherry, intranasal, safety, expression kinetics, HIV vaccines, IVIS spectrum live animal imaging

## Abstract

We have previously established that mucosal uptake of recombinant fowlpox virus (rFPV) vaccines is far superior to other vector-based vaccines. Specifically, intranasal priming with rFPV vaccines can recruit unique antigen-presenting cells, which induce excellent mucosal and systemic HIV-specific CD8^+^ T-cell immunity. In this study, we have for the first time investigated the *in vivo* dissemination, safety and expression kinetics of rFPV post intranasal delivery using recombinant viruses expressing green fluorescent protein or mCherry. Both confocal microscopy of tissue sections using green fluorescent protein and *in vivo* Imaging System (IVIS) spectrum live animal and whole organ imaging studies using mCherry revealed that (i) the peak antigen expression occurs 12 to 24 h post vaccination and no active viral gene expression is detected 96 h post vaccination. (ii) The virus only infects the initial vaccination site (lung and nasal cavity) and does not disseminate to distal sites such as the spleen or gut. (iii) More importantly, rFPV does not cross the olfactory receptor neuron pathway. Collectively, our findings indicate that rFPV vector-based vaccines have all the hallmarks of a safe and effective mucosal delivery vector, suitable for clinical evaluation.

## Introduction

Fowlpox virus (FPV; ICTV-approved acronym FWPV) is a dsDNA virus which naturally infects poultry species and is the prototypic member of the *Avipoxvirus* genus [1]. Vaccination against fowlpox disease using live-attenuated FPV was reported as early as the 1920s [2]. With the advent of recombinant DNA techniques in the 1980s, FPV was extensively used in the construction of recombinant vaccine vectors against other poultry diseases [3, 4]. Subsequently, recombinant FPV (rFPV) was trialled as a vaccine delivery vector for various human infectious diseases, most notably HIV and cancer, due to it being non-pathogenic and its ability to carry and express large amounts of foreign genetic material [5–7].

rFPV is known to infect mammalian cells with early and late gene expression and DNA replication, although virion morphogenesis and egress of infectious virus have been shown to be defective in several mammalian cell lines [4, 8]. In mice, mild pathology following intranasal (i.n.) inoculation with FPV has been reported with little or no virus replication [9]. However, a study suggested limited FPV replication in baby hamster kidney (BHK-21) cells [10], raising safety concerns for its use as a mucosal vector in mammalian species. BHK-21 cells are also the only known mammalian cell line permissive for modified vaccinia Ankara (MVA) [11], suggesting the vaccine viruses FPV and MVA which usually display abortive replication in mammalian cells may have a unique permissive replication when infecting BHK-21 cells. Further studies are required to substantiate the significance of these findings.

Systemic delivery of avian-specific canarypox virus (CNPV) and FPV vector-based vaccines has proven to be extremely safe in humans [12–14]. Similarly, MVA virus has also been shown to be safe in humans due to its abortive replication in mammalian cells [15]. The safety of aerosol-delivered recombinant MVA and recombinant New York vaccinia (NYVAC) viruses has been well documented in mice and macaques [16, 17]. However, no comprehensive analysis of *in vivo* rFPV dissemination has been determined following i.n. delivery.

We have shown that rFPV is an excellent mucosal delivery vector compared to recombinant DNA or recombinant vaccinia virus [18, 19] and that vaccine–vector combinations can generate vastly different immune outcomes in a prime-boost setting [19, 20]. These studies have also shown that mucosal immunization can recruit unique antigen-presenting cell subsets to the mucosae [21, 22] and induce high-avidity CD8^+^ T cells with better protective efficacy against HIV-1 [23]. In view of the relevance of rFPV as a candidate vector for mucosal vaccination against HIV-1, the current study investigated virus uptake via the nasal mucosa and evaluated expression kinetics, distribution and safety of rFPV expressing HIV-specific antigens together with green fluorescent protein (FPV-HIV-GFP) or mCherry (FPV-HIV-mCherry) following i.n. delivery. The latter construct was specifically used for whole organ or live animal imaging using the IVIS^TM^ Spectrum Imaging System.

## Results

### FPV-HIV vaccines co-expressing HIV antigens together with GFP or mCherry

**Fig. 1.**
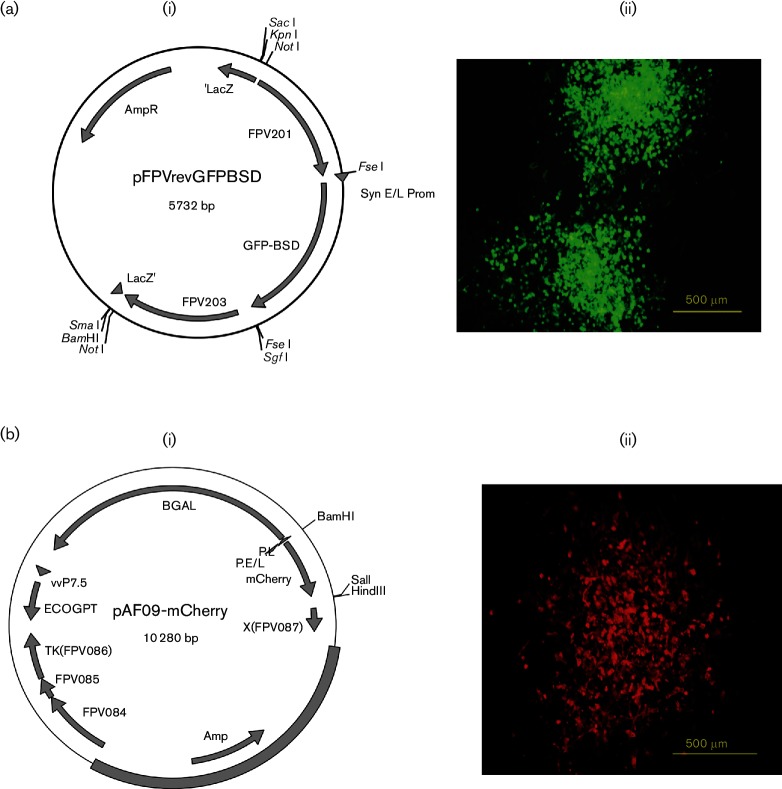
(a) Construction of rFPV-expressing GFP. (i) A custom FPV sequence was inserted into the cloning vector pUC57 made to order by GenScript. The synthetic DNA included regions of the FPV201 and FPV203 ORFs, replacing the ‘rev’ sequences with a multiple cloning site containing a unique FseI restriction site inserted between the end of ORF FPV201 and the putative promoter region of FPV203. A synthetic E/L promoter and GFP–blasticidin S deaminase (BSD) gene cassette was inserted into the FseI site in the same orientation as the FPV201 and FPV203 ORFs. (ii) The image represents rFPV plaques expressing GFP, at magnification ×10. (b) Construction of rFPV-expressing mCherry. (i) The mCherry was cloned into pAF09 plasmid, and rFPV was constructed as described in the Methods using homologous recombination. (ii) The image represents rFPV plaques expressing mCherry, at magnification ×10.

The plasmids encoding selectable markers and GFP (pFPVrev-GFPBsd) or mCherry (pAF09-mCherry) were constructed [Fig. 1a, b(i)]. Recombinant viruses FPV-HIV-GFP and FPV-HIV-mCherry were isolated by identifying recombinant plaques under fluorescence microscopy and plaque purification using standard methods [Fig. 1a, b(ii)].

### Peak antigen expression occurs at 12 to 24 h post i.n. rFPV delivery

**Fig. 2.**
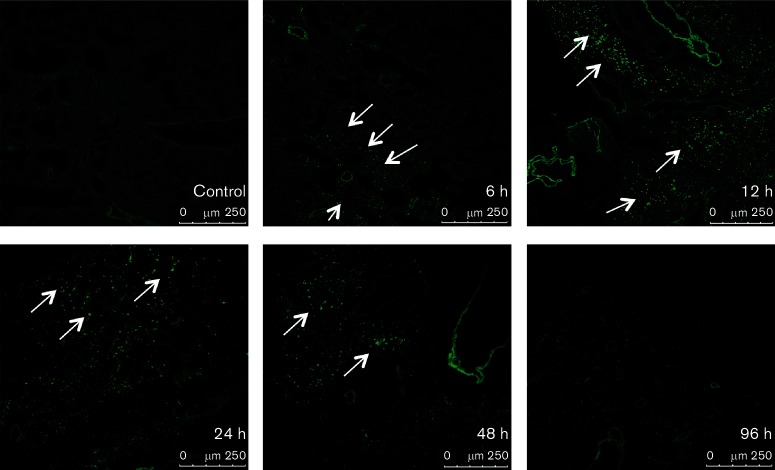
Expression kinetics of recombinant mCherry protein in the lung. BALB/c mice (*n*=3) were immunized i.n. with 2×10^7^ p.f.u. FPV-HIV GFP vaccine, and at the indicated time points post vaccination, lungs were harvested and frozen in optimum cutting temperature compound as described in the Methods. The GFP expression was analysed in 7-µm-thick sections using confocal microscopy. First image (top left) represents the fluorescence background level in unimmunized lung tissue (control), and other images represent lung tissue sections at 6, 12, 24, 48 and 96 h post FPV-HIV GFP vaccination. Each image is a representative of three animals.

Previous studies in our laboratory have established that FPV-HIV is an excellent mucosal delivery vector that induces a high-avidity HIV-specific CD8^+^ T-cell repertoire [18, 19, 21, 23]. Hence, in this study, following i.n. delivery of FPV-HIV-GFP, the kinetics of rFPV protein expression in BALB/c (H-2^d^) lung tissue were analysed at 6, 12, 24, 48 and 96 h post immunization using confocal microscopy. Results indicated that GFP expression in lung tissue was detected as early as 6 h post immunization, with expression peaking at 12 to 24 h, followed by a decline at 48 h. Interestingly, no detectable recombinant protein expression was detected 96 h post vaccination, suggesting that rFPV vector-directed gene expression was short lived (Fig. 2).

**Fig. 3.**
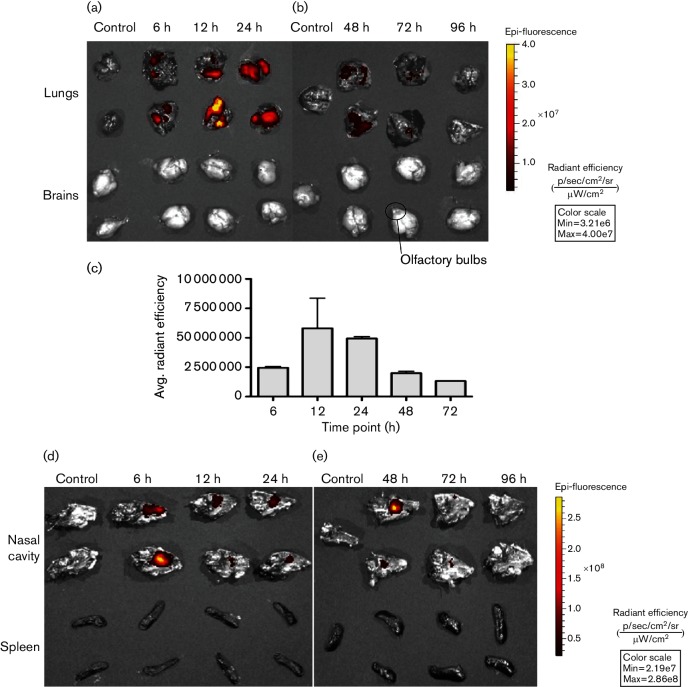
IVIS spectrum imaging analysis of lung, brain, nasal cavity and spleens following FPV-HIV-mCherry i.n. immunization. (a–e) BALB/c mice (*n*=2 per time point) were immunized with 1.5×10^7^ p.f.u. of FPV-HIV-mCherry and sacrificed at 6, 12, 24, 48, 72 and 96 h post vaccination. Lung, brain (olfactory bulb), nasal cavity and spleens were harvested and imaged with the IVIS spectrum using the following parameters: field of view, 13.4 cm; f-stop, 2; binning, 8×8; light exposure time, 5 s. Two images were generated at wavelength excitation of 500 nm and emission of 620 nm and excitation of 570 nm and emission of 620 nm, and non-specific fluorescence was recorded using the lower excitation wavelength (500 nm) and background subtraction was performed using image math analysis in Living Image Software 4.4. Production of light (photons), using an epi-fluorescence source of excitation, was quantified by radiant efficiency of light production (photons s^−1^ cm^−2^ steradian^−1^ per µW cm^−2^) highlighted in the scale bar. (c) Region of interest analysis was performed on lung samples, and average radiant efficiency was quantified (photons s^−1^ cm^−2^ steradian^−1^ per µW cm^−2^). Data represent mean±sem. (a and b) Top two rows indicate lung samples; and bottom two, brain samples. The left column in (a) indicates control lung from unimmunized mouse (top) and 12 h post i.n. FPV-HIV parental control immunized mouse (bottom). Similarly, the bottom left column indicates control brain (olfactory bulb) from unimmunized mouse (top left) and 12 h post i.n. FPV-HIV parental control immunized mouse (bottom left). The left column in (b) indicates lung (top) and brain (bottom) from unimmunized control mice. (d and e) Top two rows indicate nasal cavity samples; and bottom two, spleen samples. The left column in (d) indicates control nasal cavity from unimmunized mouse (top) and 12 h post i.n. FPV-HIV parental control immunized mouse (bottom). Similarly, the bottom left column indicates control spleen from unimmunized mouse (top left) and 12 h post parent FPV-HIV control immunized mouse (bottom left). The left column in (e) indicates control nasal cavity sample (top) and spleen (bottom) from unimmunized control mice.

**Fig. 4.**
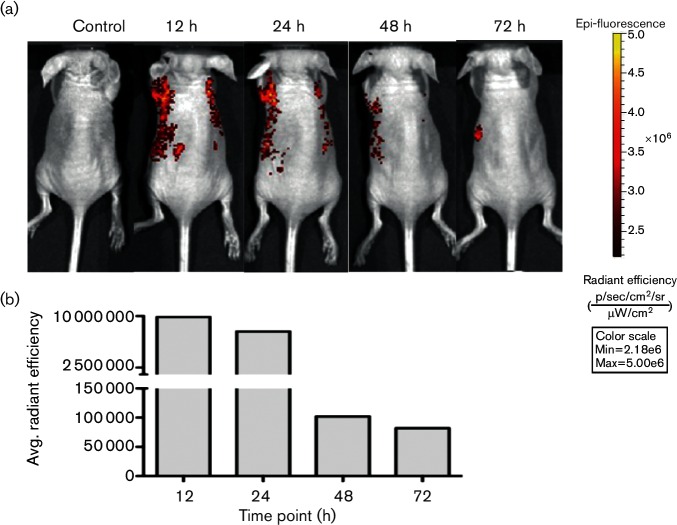
IVIS spectrum imaging of nude mice after i.n. immunization with FPV-HIV-mCherry. (a) Nude mice were i.n. immunized (*n*=3) with 1.5×10^7^ p.f.u. of FPV-HIV-mCherry and were anaesthetized prior to imaging at 6, 12, 24, 48, 72 and 96 h post vaccination. One mouse was kept as an unimmunized control (left). Mice were then imaged with the IVIS spectrum using parameters and analysis as given in Fig. 3. (b) Region of interest analysis was performed on nude mice and average radiant efficiency was quantified (photons s^−1^ cm^−2^ steradian^−1^ per µW cm^−2^).

Next, these results were further confirmed by IVIS spectrum whole organ and live animal imaging studies, using FPV expressing mCherry. Similar to the FPV-HIV-GFP study, data clearly indicated that mCherry expression was detected as early as 6 h post i.n. vaccination in the whole lung, with expression peaking at 12 to 24 h, followed by a decline in expression at 48 to 72 h with no recombinant protein expression detected at 96 h (Figs 3 and 4). Interestingly, unlike the rFPV vectors tested, the positive control vaccinia virus expressing GFP (replicative virus) showed peak protein expression at 96 h (4 days) post i.n. vaccination, both in the lung and spleen (but not in the gut) (Fig. S1, available in the online Supplementary Material).

### rFPV is a safe i.n. delivery vector

**Fig. 5.**
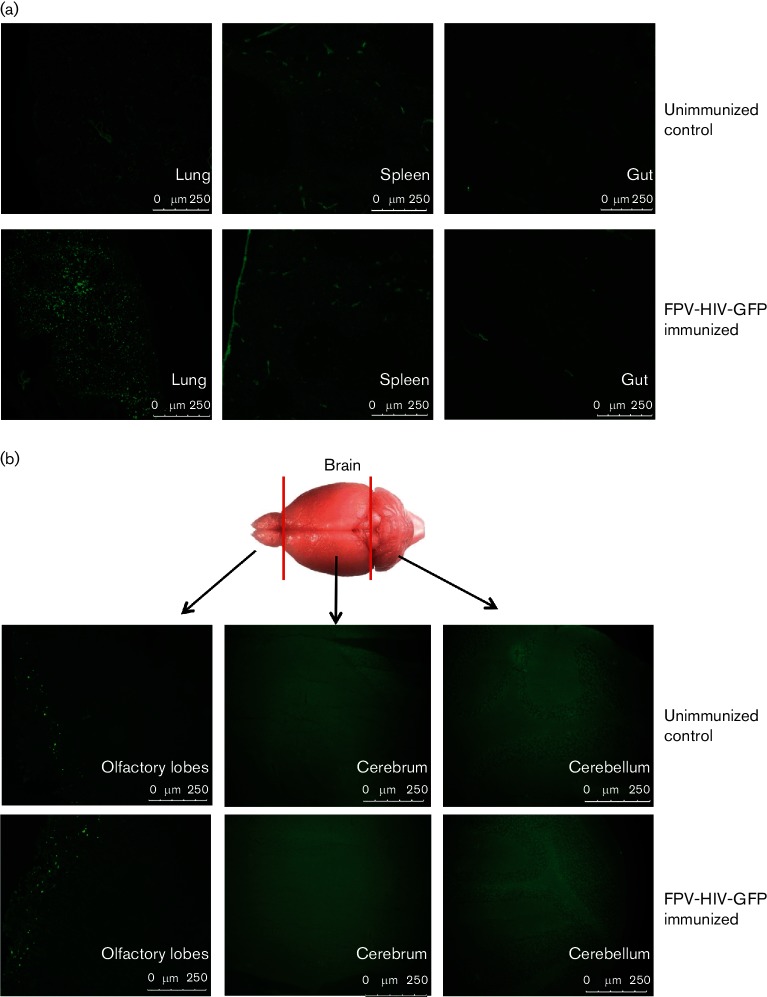
rFPV dissemination following i.n. immunization. BALB/c mice (*n*=3–4) were immunized i.n. with 2×10^7^ p.f.u. of FPV-HIV-GFP. Twenty four hours post vaccination (a) lung, spleen and gut were harvested, and GFP-expressing cells were examined as described in the Methods. The lung, spleen and gut sections obtained from unimmunized mice show the background level of fluorescence. (b) Brains were dissected, and GFP expression was analysed in three portions: olfactory bulb, cerebrum and cerebellum using confocal microscopy. The frozen sections of brain harvested from unimmunized mice represent the background level of fluorescence. Each image is a representative of three animals.

Although systemic delivery of rFPV vaccines has proven to be safe in humans [12], the safety of mucosal rFPV delivery has not yet been established. As such, rFPV dissemination following i.n. immunization was firstly evaluated in the lung, spleen, gut and brain sections of FPV-HIV-GFP vaccinated BALB/c mice as indicated in the Methods. The results demonstrated that following i.n. delivery, rFPV only disseminated to the lung compartment, but no GFP expression was detected at distal sites such as the spleen or gut (Fig. 5a). Moreover, examination of different brain sections, olfactory lobes, cerebrum and cerebellum, indicated no GFP expression (Fig. 5b).

Next, the safety and dissemination of the i.n. immunization of rFPV were further evaluated in BALB/c mice with FPV-HIV-mCherry, using *ex vivo* whole organ IVIS spectrum imaging analysis. The results indicated that rFPV only disseminated to the lung compartment (Fig. 3a, b) and nasal cavities of mice, but the expression of mCherry was much higher in the lung than nasal cavity (Fig. 3d, e). However, no rFPV-directed mCherry expression was detected at any distal sites such as the spleen (Fig. 3b, e), heart (data not shown) and importantly brain (olfactory bulb) (Fig. 3a, b). Results from FPV-HIV-mCherry immunized mice further supported the data from the FPV-HIV-GFP i.n. immunization studies, suggesting that rFPV protein expression following i.n. immunization is transient and localized to the respiratory tract and does not disseminate to other organs, specifically the brain.

### rFPV is detected in the lung only for 96 h post i.n. delivery

**Fig. 6.**
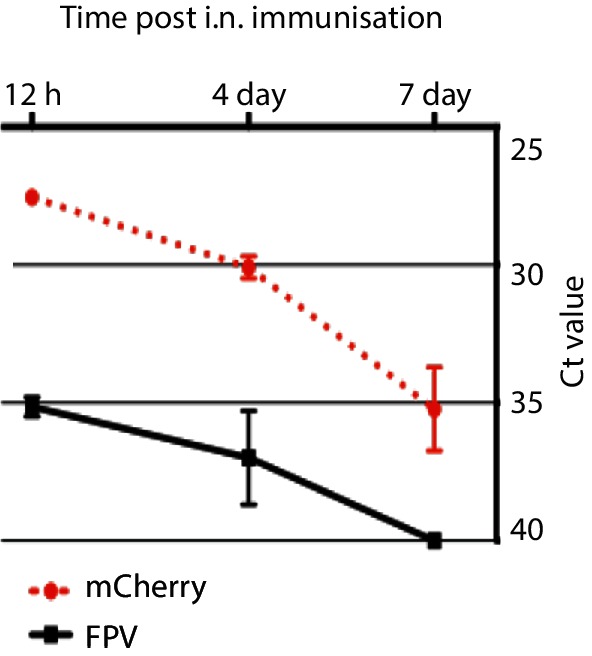
qPCR of lung samples from FPV-HIV-mCherry i.n. immunized BALB/c mice. qPCR was performed on 125 ng of cDNA from immunized BALB/c lung samples at 12 h, 4 (96 h) and 7 days post immunization using mCherry-specific primers and FPV167 ORF-specific primer sets described in the Methods. Graph indicates the mean cycle threshold (Ct) value for each time point tested. Data represent mean±sem.

BALB/c mice were immunized i.n. with 1.5×10^7^ p.f.u. of FPV-HIV-mCherry, lung and olfactory bulb (brain) samples were harvested at different time points post i.n. immunization, and cDNA was tested by quantitative polymerase chain reaction (qPCR) using mCherry and FPV167 ORF primers. No amplified product was detected with any of the olfactory bulb samples tested. However, when samples were spiked with the limiting sensitivity of FPV-HIV-mCherry (Fig. S2) within 40 cycles, amplified products were detected for every sample, further confirming that cDNA obtained from olfactory bulb tissue did not contain any material that hampered the amplification of any mCherry or FPV167 ORF cDNA. In contrast, for lung cDNA tested with mCherry primers, amplicons were detected at mean cycle threshold (Ct) values of 27.55 at 12 h, 30.1 at 4 days and 35.2 at 7 days post immunization within 40 amplification cycles. However, with FPV 167 ORF primers, amplicons were detected at Ct values of 35.15 at 12 h, 37 at 4 days and 40 or no amplification at 7 days post immunization, respectively (Fig. 6). This again indicated that, in an abortive infection, FPV early gene expression is amplified relative to late genes. Collated together, the live imaging and the qPCR data clearly indicated that although expressed protein (i.e. mCherry) mRNA persisted for up to 7 days, detectable FPV gene expression could only persist for up to 4 days (96 h) in the lung. Unimmunized olfactory bulb and lung samples showed no amplification with mCherry or FPV167 ORF primers. Positive control housekeeping gene ribosomal protein L32 was amplified in all lung and olfactory bulb samples tested including the unimmunized controls.

## Discussion

Mucosal surfaces are the first targets of infections such as HIV and tuberculosis (TB). Several studies have now clearly established that mucosally delivered vaccines offer better protective efficacy compared to systemic delivery against mucosal pathogens [23–25], suggesting that mucosal vaccine strategies may be a better option for a future prophylactic HIV-1 or a TB vaccine strategy. Although intramuscular delivery of poxvirus vectors has been shown to be safe in humans [12, 15, 26], the safety of many of these vectors delivered intranasally is poorly characterized. In the context of nasal delivery of live virus vector-based vaccines, the main concern has been the possibility of virus infection of brain tissue through the olfactory receptor neuron pathway. This has been reported with live-attenuated adenoviruses [27]. Additional studies have shown that i.n. administration of an enterotoxin-adjuvanted inactivated influenza vaccine resulted in the development of Bell’s palsy (facial paralysis) in some of the recipients [28] and was withdrawn from the market. Such studies highlight the importance of understanding the uptake and dissemination profile of pox viral vector-based vaccines such as rFPV following i.n. delivery prior to their use in clinical trials.

Following i.n. rFPV delivery, the brain tissue as well as *ex vivo* organ analysis clearly indicated that although rFPV is detected in nasal tissue from 6 to 72 h, it did not cross the blood–brain barrier, substantiating FPV’s inability to cause infection of brain tissue via the olfactory receptor neuron pathway. Also, rFPV-directed gene expression at the lung mucosae was short lived (maximum 96 h) and restricted to the route/site of inoculation. These results were highly consistent with the findings of aerosol delivery of both NYVAC and recombinant MVA in murine models and non-human primates where no vaccine-associated pathology was detected in the brain [17, 29, 30]. Alternative viral vector-based vaccines such as adenovirus vectors have shown poor immune outcomes in HIV clinical vaccine trials due to vector-specific pre-existing immunity [31]. In contrast, rFPV vaccines show no or limited pre-existing immunity in humans (similar to CNPV vectors) due to their inability to replicate in mammalian cells [4, 8, 32], suggesting that future development of poxvirus-based vaccines against chronic pathogens such as HIV-1 and TB is more promising than the other viral vector-based vaccines.

Both CNPV and FPV vectors are avipoxviruses and do not immunologically cross react with orthopoxviruses; thus they can be used in combination with other poxviruses in a heterologous prime-boost modality, circumventing the induction of vector-specific immunity [33]. Despite the fact that the phase III RV144 HIV trial demonstrated modest efficacy (31.2 %) following the administration of a CNPV-based vaccine vector (ALVAC) in addition to two glycoprotein 120 proteins (AIDSVAX) [13, 34], novel and more efficient poxvirus vector-based vaccines are required with the ability to enhance the quality/avidity of CD8^+^ T-cell immunity together with durable B-cell immunity. Prior to the RV144 trial, several studies demonstrated that CNPV-based prime-boost regimes induced poor HIV-specific IFN-γ responses (in 16 % of recipients) [35, 36]. Interestingly, it has been shown that rFPV vectors express *gag*, *pol* and *env* transgenes for longer periods and at higher levels compared to the CNPV vector-based vaccines [37]. This suggests that rFPV vectors may have better ability to induce more effective immunity toward encoded vaccine antigens. Furthermore, when compared to the FPV genome [1], the larger and more divergent genome of CNPV (with potential additional genes capable of modulating host immune responses) may contribute to the distinct immunogenicity profiles elicited by CNPV [38]. Unlike FPV and CNPV, non-avian MVA and NYVAC vectors are known to encode multiple genes for immunomodulatory functions (e.g. IL-1β binding protein and IL-18 binding protein), and studies have shown that deletion of such genes can enhance antigen-specific immunity [39–41]. Similarly, the large FPV genome also encodes genes for immunomodulatory proteins such as the IFN-γ inhibitor which binds chicken and human IFN-γ but not murine IFN-γ [42]. However, the unique FPV genes and products that have evolved to potentiate replication and dissemination in the natural avian host remain largely uncharacterized [43–45]. How these unique FPV virulence factors modulate vaccine efficacy in mammals warrants further investigation.

In the context of rFPV, we have shown that (i) it is an excellent mucosal delivery vector [6, 18, 19]. (ii) Used in a priming vaccine, it can induce unique antigen-presenting cell subsets resulting in poly-functional, high-avidity HIV-specific CD8^+^ T-cell repertoire in a prime-boost modality [18, 20–23]. (iii) rFPV has an excellent ability to package large amounts of foreign antigenic material (i.e. studies have shown that rFPV can package 65 % of the HIV genome) including immunomodulatory genes at various insertion sites [12, 18, 26, 46], highlighting the potential benefits of FPV-based vaccines against chronic infections such as HIV-1 or TB. Collectively, the current findings indicate that the site-specific localization and the transient expression profile of rFPV make it a safe i.n. (mucosal) delivery vehicle suitable for future clinical evaluation. We have also demonstrated that mCherry can be effectively used for whole organ and selective IVIS spectrum live animal imaging studies.

## Methods

### Mice and immunization

Pathogen-free 6- to 8-week-old female BALB/c (H-2^d^) mice and nude mice (BALB/c-Foxn1nu/Arc) were purchased from the Australian Phenomics Facility, the Australian National University (ANU), Canberra, and the Animal Resources Centre, Perth, respectively. Specific pathogen-free chicken eggs were purchased from the Australian SPF Services. All animals/procedures were maintained/performed in accordance with the Australian National Health and Medical Research Council guidelines within the Australian Code of Practice for the Care and Use of Animals for Scientific Purposes and in accordance with guidelines approved by the ANU Animal Experimentation and Ethics Committee (protocol no. A2014/14).

BALB/c mice were immunized intranasally under mild isofluorane anaesthesia with either 2×10^7^ p.f.u. FPV-HIV-GFP or FPV-HIV-mCherry and at 6, 12, 24, 48, 72, 96 h or 7 days post vaccination; lungs, spleen, gut, brain or nasal cavity samples were harvested to study the kinetics and dissemination of rFPV protein expression. Nude mice were immunized intranasally with 1.5×10^7^ p.f.u. FPV-HIV-mCherry and imaged using the IVIS spectrum (PerkinElmer) to study the kinetics and dissemination of rFPV protein expression in organs or in live animals.

### Construction of rFPVs expressing GFP and mCherry

To evaluate rFPV dissemination and expression kinetics post i.n. delivery in mice, plasmids containing GFP gene (pFPVrev-GFPBsd) or mCherry gene (pAF09-mCherry) were constructed. A FPV vector was constructed containing a GFP–blasticidin S deaminase (BSD) gene fusion cassette [47] modified to contain GFP mutations to enhance fluorescence signals [48] under the control of a strong synthetic poxvirus early/late promoter. The GFP-BSD cassette was inserted at the FPV ‘rev’ (reticuloendotheliosis virus) insertion site between the FPV ORFs FPV201 and FPV203, resulting in the deletion of the ‘rev’ sequence [49, 50]. A FPV vector containing the mCherry gene under the control of a strong fowlpox early/late promoter was constructed using the vector pAF09 [51, 52]. The *mCherry*, *lacZ* and *gpt* genes were inserted into the intergenic site between the FPV *thymidine kinase* gene (FPV086) and ORF FPV087 contained in the vector pAF09 [52].

Recombinant viruses were constructed by homologous recombination as described previously [50]. Parent rFPV091-HIVgagpol constructed previously [26] using the vector pKG10a [49] contains the insertion of the HIVgagpol (AE clade) genes into the intergenic region between ORFs FPV133 (VV J1R homologue) and FPV134 (J3R). Briefly, primary chicken embryo skin cells were infected with rFPV091-HIVgagpol [26] and transfected with either pFPVrev-GFPBsd or pAF09-mCherry using Lipofectamine 2000 (Invitrogen). The recombinant viruses, FPV-HIV-GFP and FPV-HIV-mCherry, were isolated after three or four rounds of plaque purification under selection. FPV-HIV-GFP was selected using blasticidin S (2 µg ml^−1^) in the medium [47]. FPV-HIV-mCherry was selected using mycophenolic acid, hypoxanthine–aminopterin–thymidine and xanthine medium [3]. Fluorescent plaques were viewed using an IX71 inverted bright-field/fluorescence microscope (Olympus). Absence of parental virus contamination was confirmed by PCR, virus stocks were prepared and viral titre was determined [50]. Viral growth curve analysis also was performed on mCherry virus. Vaccinia virus GFP containing the insertion of the GFP-BSD cassette into an intergenic site of the wild-type vaccinia virus WR strain to retain pathogenicity was used as a positive control, which was kindly provided by Dr Mayank Khanna (JCSMR/ANU).

### Tissue preparation and evaluation of GFP expression using confocal microscopy

Tissue samples extracted from intranasally immunized BALB/c mice were first washed with PBS and then rapidly frozen in Tissue-Tek^R^ optimum cutting temperature compound (ProSciTech) using solid CO_2_. Prior to freezing in optimum cutting temperature compound, brain samples were cut in half via the midline sagittal section for analysis of three major portions: olfactory bulb, cerebrum and cerebellum. Tissue samples were then stored at −80 °C until processing for fluorescence evaluation. Next, 7-µm-thick tissue sections were cut using a cryostat set at −20 °C, immediately fixed in 4 % paraformaldehyde for 5 min and washed twice with PBS. Images were then acquired using a TCS SP5 confocal microscope (Leica). In these studies, tissue samples obtained from unimmunized BALB/c mice were used as background fluorescence controls.

### Evaluation of rFPV-mCherry expression in whole organs and live animals using IVIS spectrum imaging analysis

*Ex vivo* imaging was performed on different organs harvested from intranasally immunized BALB/c mice, and live animal imaging on nude mice to evaluate rFPV-directed mCherry expression using the IVIS spectrum analysis. Live animal imaging was performed on nude mice under anaesthesia (2 % isofluorane in oxygen, XGI-8 XENOGEN Gas Anaesthesia System) as per the manufacturer’s instructions. (Note: Due to high auto-fluorescence detected with BALB/c mice, live imaging using mCherry could only be performed on nude mice.) Mice and whole organs were imaged using the IVIS spectrum under the following parameters: binning medium, f-stop 2, light exposure of 5, 10 or 15 s, field of view (C) 13.4, excitation wavelength filters of 500 and 570 nm and an emission wavelength filter of 620 nm. Fluorescence radiance efficiency and pseudocolour scales were quantified using Living Image Software 4.4 (PerkinElmer). In these studies, live unimmunized mice and organs from unimmunized and FPV-HIV immunized mice were used as background fluorescence controls.

### Evaluation of mCherry expression in the lung and olfactory bulb using real-time PCR

Real-time PCR was performed on excised lung and olfactory bulb samples obtained from unimmunized and FPV-HIV-mCherry i.n. immunized BALB/c mice 12 h, 96 h and 7 days post vaccination to evaluate the persistence and/or presence of rFPV mRNA expression. Lung and olfactory bulb samples were frozen at −70 °C prior to tissue disruption and homogenization following ‘TissueLyser LT handbook’ protocol (Qiagen). Total RNA was isolated from disrupted and homogenized samples using the ‘RNeasy Mini Handbook’ (Qiagen), analysed and quantified using the NanoDrop ND-1000 spectrophotometer, and cDNA synthesis was then performed on RNA samples using Superscript III reverse transcriptase (Invitrogen), as detailed previously [23, 53].

Primers used were mCherry (forward 5′-CCGACTACTTGAAGCTGTCCT-3′ and reverse 5′-GTAGATGAACTCGCCGTCCT3′), FPV167 ORF the major core protein 4b essential for the formation of a structurally active core (forward 5′-ACAGCAAGGATTGTGATC TTGT-3′ and reverse 5′-GCCGGTCTGAATCCTACAAT-3′) and housekeeping gene *Mus musculus* ribosomal protein L32 (forward 5′-GCTGGAGGTGCTGCTGATGTG-3′ and reverse 5′-CGTTGGGATTGGTGACTCTGATGG-3′) [23]. qPCR was performed with SYBR Green (AB) using the QuantStudio 12k Flex Real-Time PCR system (AB) at an amplification efficiency of 100 % and a thermal cycle profile of 50 °C for 2 min, 95 °C for 10 min followed by 40 cycles of 95 °C for 15 s and 60 °C for 1 min. A further step of 95 °C for 15 s, 60 °C for 1 min and 95 °C for 15 s was added for melting curve analysis (Fig. S3).

### Limiting sensitivity analysis of FPV-HIV-mCherry

From a known amount of FPV-HIV-mCherry infected primary chicken embryo skin cells with 100 % infectivity, mRNA was isolated and quantified, and cDNA was synthesized. Serial tenfold dilutions of cDNA were referenced to infected cell number as indicated in Fig. S2, and qPCR was performed with mCherry and FPV-specific primers to determine the minimum amount of cDNA that could be detected within 40 qPCR amplification cycles above the Ct (0.5 ∆RN). This determined the limiting sensitivity of cDNA that was amplified that could be used consistently as a positive spiking control reference for cDNA from unimmunized and FPV-HIV-mCherry immunized murine lung and olfactory bulb samples. This was determined as 0.033 FPV-HIV-mCherry infected chicken embryo skin cells corresponding to 0.0925 pg of cDNA (Fig. S2).

### Analysis of data

Data represent mean and sd. Region of interest analysis was performed on *ex vivo* organs and live animal images to quantify fluorescence by average radiant efficiency (photons s^−1^ cm^−2^ steradian^−1^ per µW cm^−2^) as described by the manufacturer.

## Supplementary Data

2338 Supplementary File 1
