# Supplementary material for: Recombinant fowlpox virus vector-based vaccines: expression kinetics, dissemination and safety profile following intranasal delivery
Source: J Gen Virol. 2017 Apr 1;98(3):496–505. doi: 10.1099/jgv.0.000702 (PMC5797952; doi:10.1099/jgv.0.000702)
Supplement: Supplementary File 1 [file jgv-98-496-s001.pdf]

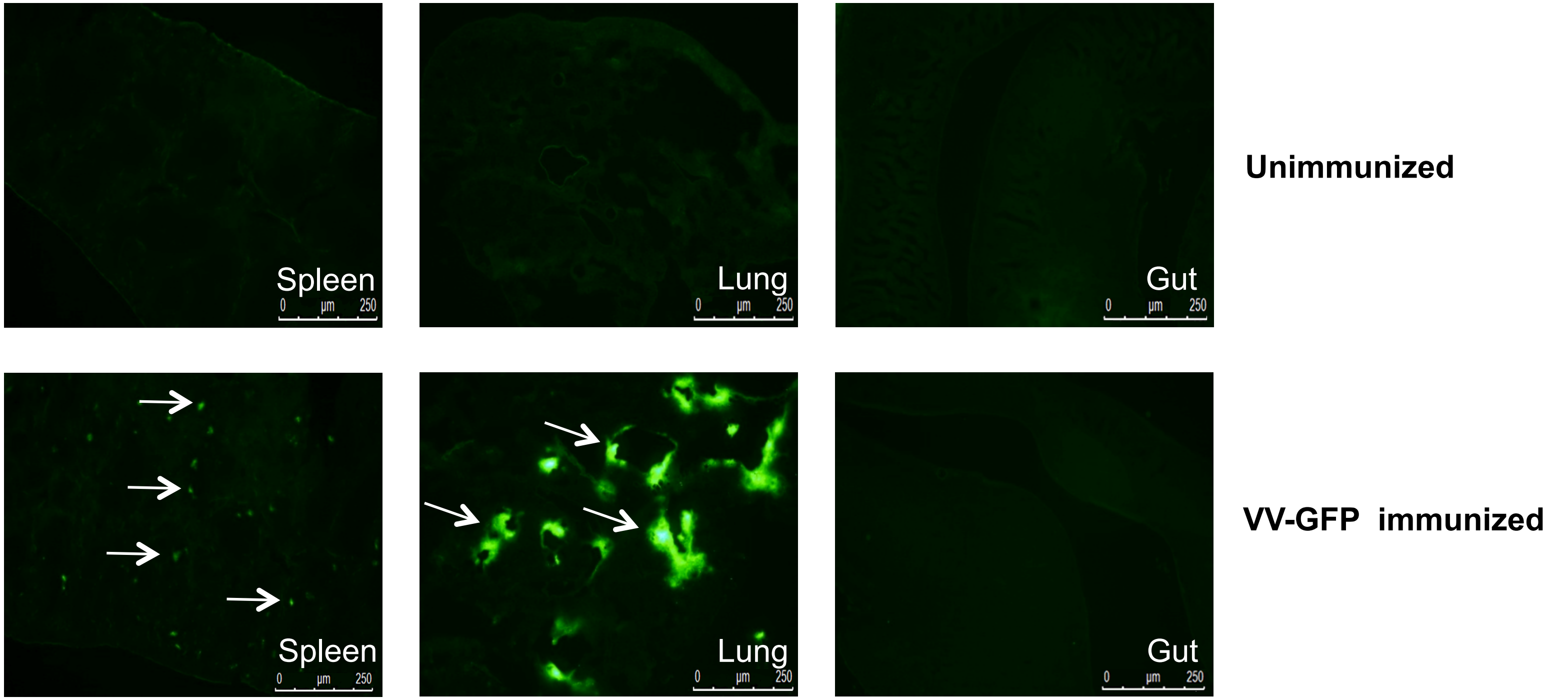

Supplementary Figure 1 in-vivo dissemination of recombinant vaccinia virus vaccine that expressed GFP (VV-GFP). (i) Images in top row represent the fluorescence background level in spleen, lung and gut tissues harvested from unimmunized mice. (ii) BALB/c mice (n=3) were immunized with 5 X 10^6 pfu of VV-GFP and 96 hours post vaccination, frozen sections of spleen, lung and gut were examined for GFP expressing cells using olympus IX71 fluorescent microscope. Following i.n. delivery, rVV disseminated in spleen and lung (arrow heads indicates the cells expressing GFP), no GFP expression was detected in gut. Each image is a representative of three animals.

(A)

cDNA dilution (pg)	Mean Ct value		Infected Cell number
	mCherry primers	FPV primers	
925	24.3	23.6	333
92.5	26.8	27.1	33.3
9.25	30.6	30.8	3.33
0.925	34.1	33.9	0.33
0.0925	36.5	37.1	0.033
	R^2= 0.9941	R^2= 0.9988	

(B)

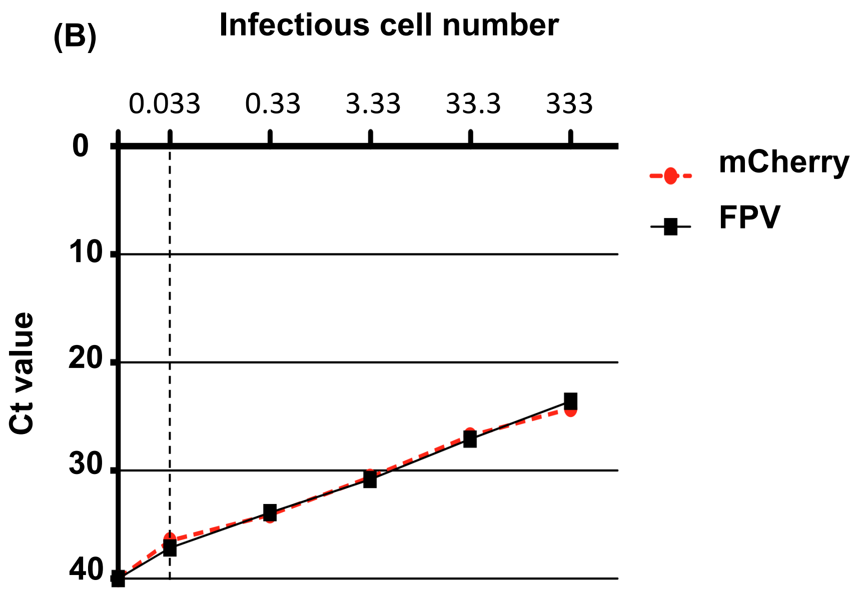

Supplementary Figure 2. Limiting sensitivity detection of FPV-HIV-mCherry infected chicken embryo skin cells using qPCR. cDNA prepared from FPV-HIV-mCherry infected chicken embryo skin cells was serially diluted ten fold and qPCR was performed using mCherry and FPV167 ORF specific primers. **Table A.** represents cDNA dilutions in pg, corresponding to infected cell numbers and the relevant cycle threshold (Ct) amplification value for each primer set. **Graph B** represents infected cell numbers against the Ct values for each primer set. R^2 values were also generated for the two sample curves for mCherry specific primers (R^2= 0.9941) and FPV167 ORF specific primers (R^2= 0.9988). Data represent mean and ± standard error of the mean. The black line represents the dilution or infected cell number of limiting sensitivity.

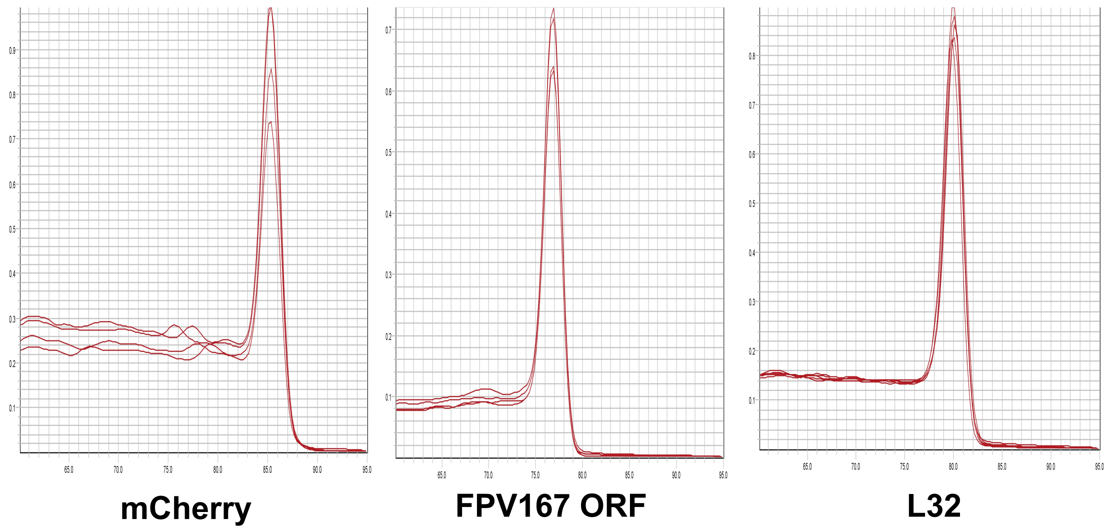

Supplementary Figure 3. Dissociation curves of the qPCR products of specific primer pairs. The dissociation curves of three specific primers for mCherry, FPV167 ORF and L32 are illustrated above. The curves above represent the peak derivative values (rate of change in fluorescence) in melting temperature of the double-stranded DNA products of each set of primer pairs.
